# Postural orthostatic tachycardia syndrome and other related dysautonomic disorders after SARS-CoV-2 infection and after COVID-19 messenger RNA vaccination

**DOI:** 10.3389/fneur.2023.1221518

**Published:** 2023-08-16

**Authors:** Elisabeth Gómez-Moyano, Jorge Rodríguez-Capitán, Daniel Gaitán Román, José Antonio Reyes Bueno, Aurora Villalobos Sánchez, Francisco Espíldora Hernández, Gracia Eugenia González Angulo, María José Molina Mora, Karl Thurnhofer-Hemsi, Ana Isabel Molina-Ramos, Miguel Romero-Cuevas, Manuel Jiménez-Navarro, Francisco Javier Pavón-Morón

**Affiliations:** ^1^Department of Dermatology, Hospital Regional Universitario de Málaga, Málaga, Spain; ^2^Centro de Investigación en Red de Enfermedades Cardiovasculares (CIBERCV), IBIMA-Plataforma BIONAND, Universidad de Málaga, Málaga, Spain; ^3^Department of Cardiology, Hospital Universitario Virgen de la Victoria, Málaga, Spain; ^4^Department of Cardiology, Hospital Regional Universitario de Málaga, Málaga, Spain; ^5^Department of Neurology, Hospital Regional Universitario de Málaga, Málaga, Spain; ^6^Department of Internal Medicine, Hospital Regional Universitario de Málaga, Málaga, Spain; ^7^Department of Neumology, Hospital Regional Universitario de Málaga, Málaga, Spain; ^8^Department of Cardiology, Hospital Quiron Salud Málaga, Málaga, Spain; ^9^Department of Computer Languages and Computer Sciences, University of Malaga, Málaga, Spain; ^10^Department of Medicine and Dermatology, University of Malaga, Málaga, Spain

**Keywords:** postural tachycardia syndrome, postural orthostatic tachycardia, POTS, postacute sequelae of SARS-CoV-2 syndrome, vaccine RNA, dysautonomia, COVID-19, SARS-CoV-2

## Abstract

The COVID-19 pandemic has caused a challenge for our society due to the post-acute sequelae of the disease. Persistent symptoms and long-term multiorgan complications, known as post-acute COVID-19 syndrome, can occur beyond 4 weeks from the onset of the COVID-19 infection. Postural orthostatic tachycardia syndrome (POTS) is considered a variety of dysautonomia, which is characterized by chronic symptoms that occur with standing and a sustained increase in heart rate, without orthostatic hypotension. POTS can lead to debilitating symptoms, significant disability, and impaired quality of life. In this narrative review, the etiopathogenic basis, epidemiology, clinical manifestations, diagnosis, treatment, prognosis, and socioeconomic impact of POTS, as well as other related dysautonomic disorders, after COVID-19 infection and SARS-CoV-2 postvaccination, were discussed. After a search conducted in March 2023, a total of 89 relevant articles were selected from the PubMed, Google Scholar, and Web of Science databases. The review highlights the importance of recognizing and managing POTS after COVID-19 infection and vaccination, and the approach to autonomic disorders should be known by all specialists in different medical areas. The diagnosis of POTS requires a comprehensive clinical assessment, including a detailed medical history, physical examination, orthostatic vital signs, and autonomic function tests. The treatment of POTS after COVID-19 infection or vaccination is mainly focused on lifestyle modifications, such as increased fluid and salt intake, exercise, and graduated compression stockings. Pharmacotherapy, such as beta-blockers, fludrocortisone, midodrine, and ivabradine, may also be used in selected cases. Further research is needed to understand the underlying mechanisms, risk factors, and optimal treatment strategies for this complication.

## 1. Introduction

The post-acute sequelae of COVID-19 represent a challenge for patients and their doctors. Although the therapeutic management and short-term consequences of the COVID-19 infection are well-known, there is less information on the persistent symptoms in patients who experience long-term multiorgan complications, namely “long COVID-19”, “chronic COVID syndrome”, “long-haul COVID”, “post-acute sequelae of SARS-CoV-2 infection”, and “post-acute COVID-19 syndrome (PACS)” (1). PACS is defined as a syndrome characterized by persistent symptoms and/or delayed or long-term complications beyond 4 weeks from the onset of COVID-19 (2–4).

The neurological complications of COVID-19, assessed collectively, represent a problem of greater magnitude for several reasons, including their high frequency, the high proportion of cases with severe neurological involvement, and their strong association with an adverse prognosis (5). Survivors with chest pain and dyspnea may have autonomic dysfunction, and the first confirmed cases of dysautonomia after COVID-19 infection have been published (6, 7). The autonomic nervous system (ANS) maintains biological homeostasis at rest and in response to stress through a complex network of central and peripheral neurons (8, 9). Postural orthostatic tachycardia syndrome (POTS) is a form of dysautonomia characterized by chronic symptoms (>6 months) that occur with standing, a sustained increase in a heart rate of 30 beats per min (bpm) or a heart rate of > 120 bpm when moving from a supine to a standing position, without orthostatic hypotension (10). One of the potential mechanisms for the development of this POTS is inadequate vasoconstriction, which is triggered by orthostasis (assuming an upright position). Excessive compensatory tachycardia and increased plasma noradrenaline levels contribute to debilitating symptoms that can lead to significant disability and impaired quality of life (11, 12). Because the ANS innervates all organs, the approach to autonomic disorders should be known by all specialists in different medical areas.

In this narrative review, we will approach the etiopathogenic basis, epidemiology, clinical manifestations, diagnosis, treatment, prognosis, socioeconomic impact of POTS, and other related dysautonomic disorders after COVID-19 infection and SARS-CoV-2 postvaccination.

## 2. Materials and methods

**Figure 1:**
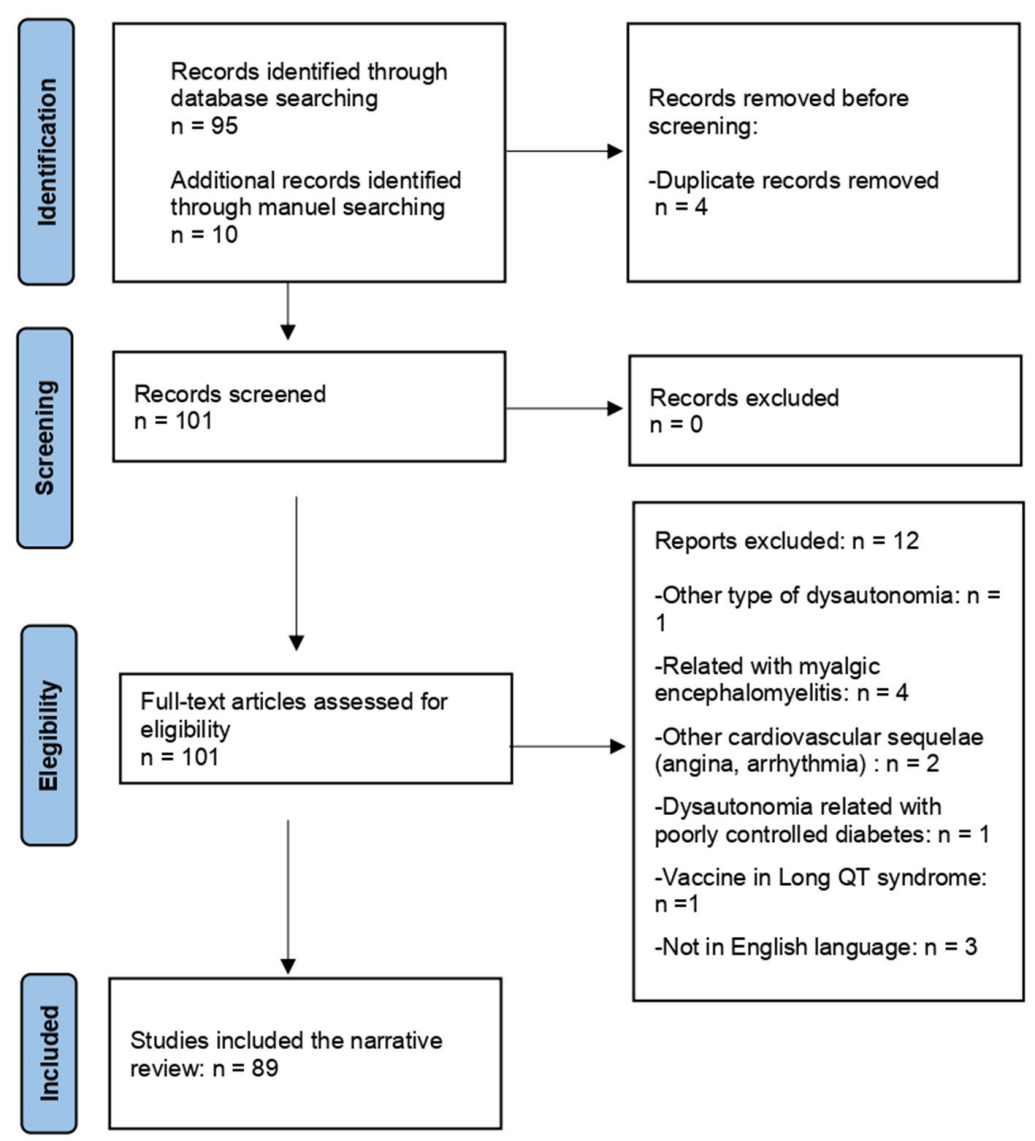
Flow chart of the narrative review.

We conducted a bibliographic search in Google Scholar, PubMed, and Web of Science databases using the keywords: (SARS-CoV-2 OR COVID-19 OR SARS-CoV-2 vaccine OR COVID-19 vaccine) AND (postural orthostatic tachycardia). We applied the following filters: free full text, case reports, clinical studies, multicenter studies, observational studies, humans, systematic reviews, and meta-analyses sorted by the most recent method. A manual selection was performed based on bibliographic references. A total of 89 relevant articles were selected. The flow chart of the search strategy is shown in Figure 1. The search was conducted in March 2023.

## 3. Etiopathogenic basis

Orthostatic intolerance is the inability to tolerate the upright posture because of sympathetic activation or cerebral hypoperfusion, which is relieved with a supine position. In orthostatic intolerance, the release of norepinephrine and epinephrine causes exacerbated tachycardia, with palpitations, chest pain, and breathlessness. Very high catecholamine levels can lead to paradoxical splanchnic vasodilatation (resulting in decreased venous return), sympathetic activity retirement, and the activation of the vagus nerve, giving rise to hypotension, dizziness, or syncope (13). The correlation between endothelial function and ANS, as well as vasomotor activity, is another major component of dysautonomia (14). Orthostatic intolerance syndromes include neuromediated syncope, neurogenic orthostatic hypotension, and POTS. The pathophysiological mechanisms resulting in autonomic dysfunction in POTS may include sympathetically mediated vasoconstriction in the lower limbs (neuropathic POTS), excessive cardiac sympathoexcitation response (hyperadrenergic POTS) with increased serum norepinephrine, volume dysregulation (hypovolemia), baroreceptor dysfunction, peripheral neuropathy, and autoimmunity due to molecular mimicry following infections by pathogens such as Epstein–Barr virus, cytomegalovirus, or Borrelia Burgdorferi (1, 15). There are several medical conditions associated with the development of POTS, such as joint hypermobility, mostly Ehler Danlos type III, and Marfan syndrome.

Numerous pathophysiological mechanisms have been proposed for autonomic impairment caused by SARS-CoV-2: immune dysregulation, hormonal disturbances, invasion of the virus into the brain, elevated cytokine levels due to immune reactions leading to chronic inflammation, direct tissue damage, endotheliitis, microthrombosis (16), and persistent low-grade infection (17).

It is not yet known whether the pathophysiology of POTS prior to the pandemic is the same as that found in patients with long COVID-19 and POTS (11, 18). Some authors have proposed that the SARS-CoV-2-generated antibodies cross-react with components of the autonomic ganglia, autonomic nerve fibers, G-protein-coupled receptors, or other neuronal or cardiovascular receptors, leading to the dysfunction of the ANS (6, 19). This hypothesis gained strength after the elevation of autoimmunity and inflammation markers was observed in patients with POTS (20, 21).

SARS-CoV-2 can invade the central nervous system through two mechanisms: the retrograde neuronal pathway or the hematogenous route. In the former, viral particles could migrate from the cribriform plate through the olfactory tract (22). The existence of this transneuronal route has been supported by studies on necropsies, which have shown a greater quantity of SARS-CoV-2 viral particles and the damage caused by them in the olfactory bulb compared to what is observed in the brainstem (23, 24). The hematogenous route is explained by the development of a disrupted blood–brain barrier, which becomes more permeable through the action of inflammatory cytokines and monocytes (25). Although this activity on the blood–brain barrier has not been demonstrated, there is evidence to support it, primarily using choroid plexus organoid models that abundantly expressed ACE2; as a consequence SARS-CoV-2 activity, tight junction integrity was compromised, resulting in cerebrospinal fluid leakage (26, 27). The viral neuronal damage can be mediated by a virus protein binding to the endothelial acetylcholine receptor or by a cytokine-mediated dysimmune mechanism (28).

The presence of endothelial dysfunction in POTS has been observed in small patient series, which supports the pathophysiological role of such dysfunction. Therefore, Chopoorian *et al*. compared 19 POTS patients with 9 healthy controls. POTS patients exhibited a lower percentage of brachial flow-mediated dilation, reflecting endothelial dysfunction in conduit arteries (29). SARS-CoV-2 disrupts the renin–angiotensin–aldosterone system, upregulates angiotensin 2, and produces proinflammatory cytokines, with sequelae on the vascular endothelium (30). SARS-CoV-2 has a tropism for the ventrolateral medulla and the nucleus tractus solitarius, where the angiotensin 2-converting enzyme receptor is highly expressed (31).

One proposed but poorly recognized etiopathogenic mechanism of neurological damage secondary to SARS-CoV-2 infection is the increase in the concentration of neuroinflammatory biomarkers, primarily neurofilaments and total tau. The evidence supporting this mechanism is stronger in patients with severe COVID-19 and central neurological involvement, but an elevation in blood concentration of neurofilaments has also been observed in cases with mild neurological involvement or even without neurological symptoms (32, 33).

## 4. Epidemiology

The estimation of the prevalence of POTS and related autonomic disorders after SARS-CoV-2 infection is particularly challenging for various reasons, similar to many other manifestations associated with long COVID-19. First, clear temporal boundaries between SARS-CoV-2 infection and the development of autonomic disorders have not been described, making it difficult to establish a clear association between the two in each individual patient. Additionally, the high prevalence of SARS-CoV-2 infection makes it unfeasible to adequately select patients unexposed to such an infection in potential case–control studies.

A retrospective Spanish study of 841 hospitalized COVID-19 patients showed that the prevalence of autonomic dysfunction was 2.5% (3). It is estimated that 2.15–6.45 million people across the world have suffered from post–COVID-19 dysautonomia (34). A study of 60 patients hospitalized with severe or critical COVID-19 actively evaluated the presence of orthostatic hypotension or POTS at the time of discharge, revealing frequencies of 48.3% and 16.7%, respectively. At 2 months post-discharge, symptoms had resolved in 89.7% of patients with orthostatic hypotension and 80% of POTS patients (35).

Autonomic dysfunction post–SARS-CoV-2 infection affects primarily female patients (80%) (2) without pre-existing conditions and a wide age distribution (36), with POTS being extremely rare among either prepubertal girls or postmenopausal women, suggesting a possible role for sex hormones. In a recent systematic review of cardiovascular autonomic dysfunction and COVID-19, POTS was the most frequent diagnosis in individuals with post–COVID-19 orthostatic complaints (37). In another series of 70 patients with persistent symptoms, most of them met the criteria for cardiovascular autonomic dysfunction (38). In a series of 28 patients presenting to a dysautonomia clinic with persistent neurologic and cardiovascular complaints after COVID-19, 20 patients had evidence of new orthostatic intolerance defined by the tilt test or 10-min stand test; 70% of them were women, and 80% required pharmacological treatment (20). The timing of POTS symptom onset was concurrent with usual acute COVID-19 symptoms, but it may occur months after infection (39, 40).

## 5. Clinical manifestations

POTS is the most common cause of chronic orthostatic intolerance, and it is characterized by an abnormal heart rate increase on standing, without orthostatic hypotension. According to the definition of the clinical condition, the most frequently observed symptoms in POTS associated with SARS-CoV-2 infection belong to the cardiovascular spectrum, with palpitations and chest pain being the most commonly described; other cardiovascular-related symptoms that have also been reported include exercise intolerance, fatigue, and vasovagal syncope (41, 42). In addition to these symptoms with a higher cardiogenic profile, POTS associated with SARS-CoV-2 infection has been reported to present symptoms from various other spheres, including neurological (headache/migraine, mental clouding, cognitive impairment, concentration problems, anxiety, light/sound sensitivity, blurred/tunnel vision, dizziness, neuropathic pain, and sleeping disorders), musculoskeletal (muscle weakness or pain), gastrointestinal (nausea, constipation, diarrhea, abdominal pain, and weight loss), respiratory (hyperventilation, asthma, and shortness of breath), urogenital (bladder dysfunction, nocturia, and polyuria), and cutaneous (petechiae, rash, erythema, abnormal sudomotor regulation, pallor, flushing, and BASCULE syndrome, which is defined as bier anemic spots, cyanosis, and urticaria-like eruption) (17, 41, 43–46). A clinical manifestation frequently described in these patients has been exercise intolerance. It shows a multifactorial origin, but a common mechanism has been proposed: sympathetic predominance and decreased response to parasympathetic and sympathetic stimuli that alter muscle tone and cardiovascular function (47). Patients often develop post-exercise malaise or flare due to vasomotor dysregulation during exercise. Systemic deficits can be maintained for only weeks but can potentially cause long-term incapacity (48).

Furthermore, certain cases of POTS associated with SARS-CoV-2 infection have been reported, which showed other cardiovascular complications apparently related to COVID-19 and not directly to POTS. Thus, the presence of intermittent left bundle branch block with septal flash on echocardiogram (49), cardiomyopathy (50), or leg ischemia with thrombus at the suprarenal aorta associated with hyperhidrosis have been described (51).

Patients who develop long COVID-19, regardless of the presence or absence of POTS, often experience neurological symptoms characterized by alterations in memory, information processing speed, difficulties concentrating, and dysexecutive disorders (52). Thus, they exhibit a clinical profile very similar to that experienced by patients with neurological disorders associated with POTS. In the clinical experience of the authors of this review, a distinguishing feature of neurological symptoms secondary to POTS, as opposed to other types of patients with long COVID-19, is the improvement of symptoms upon assuming a supine position.

## 6. Diagnosis

The diagnosis of autonomic disorders requires a meticulous medical history because symptoms are multiple and non-specific. When POTS is suspected or in the presence of inconclusive but compatible symptoms, it is highly valuable to assess heart rate using an active standing test (NASA Lean Test) and a head-up tilt test (53–56). Heart rate and blood pressure should be measured after 5 min of lying supine, and measurements should be repeated after 2, 5, and 10 min of standing to determine the presence of tachycardia and/or hypotension. The diagnostic criteria of POTS are a sustained increase in a heart rate of 30 bpm when moving from a recumbent position to a standing posture (or 40 bpm for age 12–19 years) and tachycardia of more than 120 bpm, within the first 10 min of standing, in the absence of orthostatic hypotension (a 20-point drop in systolic blood pressure). During the assessment, it is essential to inquire about symptoms and carefully observe for the presence of acrocyanosis, a purple discoloration of the extremities that commonly manifests in approximately 50% of affected individuals (57). Notably, the magnitude of heart rate variations observed during the active standing test is considerably smaller when compared to those observed during the head-up tilt test. This disparity can be attributed to the contraction of the gastrocnemius muscle during the active standing test, which leads to an increased cardiac preload (58). Nitroglycerin provocation is a standard part of the head-up tilt test protocol for assessing vasovagal syncope that increases the sensitivity for orthostatic intolerance symptoms but may lower the specificity for the reproduction of clinical symptoms (59, 60). A high value of heart rate variability indicates a healthy autonomic and cardiovascular response, while a low value is suggestive of improper coordination between sympathetic and parasympathetic systems to provide an appropriate heart response (61, 62). Tilt tests are especially useful in individuals with inappropriate sinus tachycardia, orthostatic tachycardia, unexplained syncope, or syndromes of orthostatic intolerance.

In cases where the diagnosis of POTS has not been definitively established through an active standing test or a head-up tilt test, the performance of other autonomic function tests, such as the Valsalva maneuver and deep breathing, should be considered. In the context of orthostatic intolerance, the Valsalva maneuver is considered positive due to the inability to generate secondary hypertension following initial hypotension. The deep breathing test will be positive if it triggers an exaggerated sinus tachycardia (36). An abnormal test is considered a diagnosis of cardiovascular autonomic dysfunction, and two or more abnormal tests for the diagnosis of cardiovascular autonomic neuropathy (63). The assessment of sudomotor function is another diagnostic strategy to consider in selected patients, as it has demonstrated its utility in evaluating dysautonomia in individuals with post–COVID-19 sequelae (64, 65). Sudoscan assesses sudomotor function, an indirect index of sympathetic cholinergic non-myelinated C-fiber activity since sweat glands lack parasympathetic innervation (65).

The measurement of inflammation or autoimmunity markers is a reasonable option in the context of clinical suspicion of POTS associated with SARS-CoV-2 (41, 66). First, there is increasing evidence regarding the pathophysiological role of autoantibodies against alpha and beta-adrenergic and muscarinic receptors in POTS not related to SARS-CoV-2, although the proposed pathological mechanisms have not yet been fully demonstrated (13, 67–71). Additionally, we must consider the numerous reported cases of POTS associated with SARS-CoV-2, in which the elevation of inflammatory markers or autoimmunity has been detected. In a series of 29 patients with symptomatic post–COVID-19, all developed antibodies directed against different G-protein-coupled receptors (alpha adrenoreceptor, beta-adrenoreceptor, angiotensin II, nociceptin, and muscarinic) known to be able to disturb the balance of neuronal and vascular processes (72). Other autoantibodies in POTS include ganglionic neuronal nicotinic acetylcholine receptor (g-AChR), circulating anti-nuclear, anti-thyroid, anti–NMDA-type glutamate receptor, anti-opioid like-1 receptor, anti-cardiac protein, anti-phospholipid, and Sjögren's antibodies (36, 73). Levels of G-AChRs antibody titers >1.0 nmol/L are quite specific for autoimmune autonomic ganglionopathy (74).

Complete blood count, albumin, renal function, electrolytes, B-type natriuretic peptide, thyroid stimulating hormone, and morning cortisol should be evaluated in all patients with suspected or diagnosed POTS to exclude other causes of tachycardia and assess relevant comorbidities. Hyperalbuminemia is an important clue suggesting hypovolemia that can be present in patients with dysautonomia (73). Holter ECG monitoring is a test that also holds great value in the diagnosis of POTS and serves as a screening tool for other related pathologies. Other diagnostic tests that can provide value in ruling out potentially relevant comorbidities, and therefore may be indicated in certain patients, include 24 h ambulatory blood pressure monitoring, chest X-ray, echocardiography, chest computed tomography, cardiac magnetic resonance, and exercise testing (75–77). The 6-min test may be indicated in certain cases with significant impairment (36). Patients with POTS following post COVID have been reported with peripheral oxygen desaturation during a 6-min walking test (41), platypnea orthodeoxia (78), or declined end tidal carbon dioxide, while the patient was in the supine position and head-up (79). These features have not been observed in POTS pre-pandemic, and they can explain why many patients with POTS following post COVID remained highly symptomatic despite multiple treatment modalities. The composite autonomic symptom scale 31 questionnaire (COMPASS 31) is a valuable tool validated to perform a comprehensive assessment of dysautonomic symptoms: vasomotor, secretomotor, gastrointestinal, urinary, and pupillomotor symptoms, as well as orthostatic intolerance. This scale has been extensively validated in other pathologies such as multiple sclerosis (80), and recently, it has been used in the diagnosis of dysautonomia in patients with a recent prior diagnosis of COVID-19 (81).

In the differential diagnosis of POTS, it is important to exclude other causes of sinus tachycardia such as dehydration, hyperthyroidism, other viral infections, pulmonary embolism, cardiac disease, anxiety, anemia, metabolic disorders, deconditioning, or chronic fatigue syndrome (82). Chronic fatigue syndrome and POTS share a hemodynamic and neurohormonal profile (83).

## 7. Treatment

The treatment of POTS has traditionally been classified into non-pharmacological measures and pharmacological measures. The effectiveness of all these strategies is limited, leading to many patients developing chronic disorders despite multiple therapeutic interventions.

In the case of POTS related to SARS-CoV-2 infection, published cases have supported the recommendation of various non-pharmacological interventions, such as increased salt and fluid intake (unless contraindicated), waist-high compression garments, and regular and graduate-supervised exercises (84, 85). The utility of an exercise program in a recumbent or semi-recumbent position (rowing or cycling) for 8 weeks, followed by a subsequent 4-week jogging phase, has been demonstrated (86). The role of physical activity including exercise in severely exercise-intolerant patients remains unclear and should be undertaken with great caution (11). It is advisable to avoid situations that can exacerbate symptoms, which has also been shown to be beneficial in POTS after SARS-CoV-2 infection (Table 1) (17, 87, 88).

**Table 1:** Therapeutic management of POTS.

**Non-pharmacological treatments (10, 13, 83, 87–90)**
Water intake: 2–3 l/day Oral NaCl intake: 10–12 g/day 20–40 mm Hg compression garments; focus on the abdomen and legs Sleep in a head-up tilt position (>10°) Drinking water before getting up in the morning Exercise training: semirecumbent with simple isometric, aerobic, and resistance exercises (84) Avoid situations that may exacerbate symptoms: sleep deprivation, exposure to heat or hot environments, alcohol intake or large meals, prolonged standing, anemia, and dehydration (17) Educate patients that POTS is a dynamic disease and that infections can significantly impact the disease trajectory (128) Moving carefully from a lying or sitting to a standing position (129) Counterpressure maneuvers: crossing legs (130) and squeezing thigh muscles, clenching buttocks, and tightly folding arms are useful to activate the skeletal muscle pump to increase venous return and prevent syncope Small frequent meals and fewer refined carbohydrates are recommended for glycemic balance and to avoid postprandial hypotension (131) It is advisable to regulate activity to achieve adaptive goals and to reduce the severity of flares and fatigue (11)
**Pharmacological treatments** (91–103)
Fludrocortisone 0.1–0.2 mg/daily Desmopressin 0.1–0.2 mg Erythropoietin 10,000 IU/weekly Propranolol 10–20 mg up to 4 daily Ivabradine 2.5–7.5/12 h Pyridostigmine 30–60 mg up to 3 daily Midodrine 2.5–15 mg 3 per day Octreotide 10–30 mg intramuscular Droxidopa 100–600 mg/8 h Methyldopa 125–250 mg/12 h Clonidine 0.1–0.2 mg orally o patch Intravenous immunoglobulin and plasmapheresis
**Non-invasive neuromodulation** **(****104**, **105****)**

Non-pharmacological measures are the first step in the treatment of POTS and dysautonomic disorders after SARS-CoV-2 infection; however, in most cases, these will be insufficient, necessitating the prescription of pharmacological treatment. The evidence regarding pharmacological efficacy in this scenario is limited and derived from small observational studies. Therefore, it is recommended that pharmacological treatment be guided by the patient's hemodynamic profile: beta-blockers, ivabradine, and metoprolol for tachycardic phenotype; midodrine, pyridostigmine, and droxidopa for hypotensive phenotype; fludrocortisone and desmopressin for hypovolemic phenotype; and clonidine and methyldopa if hyperadrenergic features with hyperhidrosis and tachycardia (36, 88, 89). A small case series of POTS patients following COVID-19 have reported symptomatic improvement associated with treatment using ivabradine (90, 91) and midodrine (91). In a cohort of 17 patients with post–covid autonomic dysfunction, 80% of those treated with beta-blockers reported improved or resolved symptoms, while 20% reported unchanged symptoms; one patient on midodrine reported improvement in symptoms, while one patient receiving colchicine reported unchanged symptoms (2). We must remember that colchicine has been reported to be selectively toxic to cholinergic neurons (92). Other pharmaceutical options for POTS after SARS-CoV-2 infection include intravenous saline, verapamil (93), and omega-3 fatty acid supplementation (94, 95). As a complementary pharmacological strategy, it is recommended to discontinue the intake of noradrenaline reuptake inhibitors (such as duloxetine, nortriptyline, and tapentadol) and avoid drugs that exacerbate orthostatic intolerance (dihydropyridine calcium channel blockers, diuretics, nitrates, and opiates) (96).

The literature describes cases of patients who remain highly symptomatic despite the previously described basic pharmacological and non-pharmacological measures. Multiple therapeutic strategies have been reported for these cases, most of which have limited evidence regarding their outcomes. We will now proceed to describe those strategies that have been tested in the context of POTS following SARS-CoV-2 infection. The stellate ganglion block has shown improvement in symptoms of long COVID-19 (97). Other therapeutic options include non-invasive neuromodulation such as vagus nerve stimulation, transcranial direct current stimulation, and repetitive transcranial magnetic stimulation (98). Recently, interest has grown in the use of cardiac neuromodulation in POTS (99). One patient has been reported under treatment with enhanced external counterpulsation (100). Cognitive behavioral therapy (101, 102), breath retraining, and paced postural exercises incorporating breathing, such as yoga and hyperbaric oxygen, have been proposed (103–105). The increased demand for autonomic specialists and clinics since the start of the COVID-19 pandemic reveals the need for more resources to adequately care for this patient population (106).

## 8. Prognosis

A 2019 study of long-term outcomes from China demonstrated that 48.4% of pediatric patients with POTS were free of symptoms at the 1-year follow-up, with 85.6% being symptom-free after 6 years (107). However, it is unclear how the treatment of these long COVID-19 patients and their prognoses may differ from other cases of classic POTS pre-pandemic (2). The duration of POTS related to COVID-19 remains unknown.

A relapse in symptoms after a COVID-19 reinfection has been described (39). The rate of long COVID-19 in those who recovered from a first infection but developed long COVID-19 following reinfection and the impact of reinfection on those with pre-existing long COVID-19 are crucial to understand to inform future policy decisions (108). Similar to long COVID-19 (109), POTS severity can fluctuate unpredictably, making rehabilitation and return to work challenging (11). It would be interesting to study whether the fluctuations may be related to new exposures to SARS-CoV-2.

Several studies on long COVID-19 suggest that patients with postural orthostatic intolerance may be further along the path of clinical recovery than those demonstrating POTS (110). Although cerebral blood flow improves with time, it remains abnormal (44, 45). Many uncertainties persist regarding the perioperative risk of COVID-19 survivors (111).

## 9. Socioeconomic impact

According to data from an online survey of people with suspected and confirmed COVID-19 infection, at 7 months after suspected or confirmed COVID-19 infection, 45.2% of patients required a reduction in working hours due to residual symptoms, and 22.3% were not working due to illness (112).

In a series of 20 patients with persistent cardiovascular and neurologic symptoms, 85% of them had residual autonomic symptoms, with 12 (60%) unable to return to work despite adequate treatment after 6–8 months (20).

The socioeconomic impact of symptom persistence after COVID-19 infection is due to autonomic and neurohemodynamic involvement, revealing the need for early intervention. Better recognition of autonomic dysfunction could reduce morbidity and socioeconomic impact (17). According to this, NICE recommends that long COVID-19 services should be led by a doctor, with the skills to diagnose POTS and exclude conditions with similar symptoms (8). The American Autonomic Society highlights that addressing the needs of patients with long COVID-19 and POTS will take a significant investment of funding and resources, both for clinical care and investigation, to allow for reducing the burden of symptoms in these patients, while acting during their window of opportunity (113). In addition to this, multidisciplinary teams are necessary (114). It is important to educate healthcare professionals to recognize complications and conditions arising from COVID-19, such as POTS (115).

## 10. POTS and vaccination

Vaccination with effective tools is crucial to control the COVID-19 pandemic and reduce its burden (30). However, severe adverse reactions have been reported following the COVID-19 vaccine, including myocarditis/pericarditis, thrombotic events, rare cases of arrhythmia, hypertension, acute coronary syndrome, and cardiac arrest (30, 116).

In a cohort of 2,84,592 COVID-19-vaccinated individuals, using a sequence–symmetry analysis, the odds of receiving the diagnosis of POTS 90 days after vaccination compared with 90 days before vaccination was 1.33 (1.25–1.41). The risk for POTS-related diagnoses was 5.35 (5.05–5.68) times higher after SARS-CoV-2 infection than after vaccination. The authors conclude that the diagnosis of POTS after vaccination might be reaching a higher frequency than expected, although this frequency would be lower than that observed after SARS-CoV-2 infection (117–119).

To date, we have identified 12 reported cases of patients diagnosed with new-onset POTS 2–21 days after receiving COVID-19 vaccines (Table 2) (74, 79, 120–125). Sex distribution was similar, and 66% of reported patients with POTS after the vaccine presented with unremarkable medical histories. Interestingly, half of the 12 reported patients had serum markers of possible autoimmunity. One patient developed severe autonomic failure with α3-ganglionic acetylcholine receptor antibodies (124). It has been proposed that SARS-CoV-2 antibodies may cross-react with the receptors in the ganglia.

**Table 2:** Reported cases of POTS related to vaccine COVID-19.

**Reference**	**Age/sex**	**Dose/timing**	**Medical history**	**Marker of autoimmunity**
Reddy et al. (120)	42/male	First/6 days	Hypothyroidism and B12 deficiency	Unknown
Hermel et al. (79)	46/female	First/48 h	Allergic rhinitis and COVID-19 infection 2 months earlier	Unknown
Park et al. (121)	40/male	First/7 days	Healthy	Negative
Sanada et al. (122)	13/male	Second/1 day	Healthy	Negative
Maharaj et al. (123)	15/male	Second/14 days	Healthy	Unknown
Rowe et al. (124)	56/male	Second/5 days	Hypertension	+ alpha-3 ganglionic acetylcholine receptor
Karimi Galougahi et al. (125)	29/male	First/4 days	Healthy	ANA+
Eldokla et al. (74)	37/female	First/7 days	Seasonal allergy and depression	Negative
	21/female	First/12 days	Healthy	+ ganglionic-acetylcholine receptor
	46/female	First/14 days	Healthy	+ Peroxidase antibody
	19/female	Second/18 days	Healthy	ANA+, IL-10, factor necrosis tumor alpha
	17/female	Second/21 days	Healthy	Elevated Il-2, Il-10, Il-13

In a cohort of 267,515 individuals who received the third and 32,934 individuals who received the fourth COVID-19 vaccine dosage at the vaccination center in Japan, 0.01–0.1% present vasovagal syncope/presyncope (126). On the other hand, the relationship between long COVID-19 and vaccines remains unclear. A study conducted in the United Kingdom on 6,030 participants showed that the odds of long COVID-19 symptoms, including autonomic dysfunction, were reduced by almost half after receiving the two doses of the SARS-CoV-2 vaccine (127). In a cohort of six post-acute COVID-19 patients with dysautonomia, 50% reported an improvement in symptoms after vaccination, while 50% reported no difference in symptoms.

The benefit of COVID-19 vaccine administration still outweighs the risk of cardiovascular and neurologic adverse reactions. However, healthcare workers must know about potential comorbidities during the vaccination period. More studies are needed to investigate the incidence of POTS occurring after COVID-19 vaccination and to demonstrate a possible causality (117).

## 11. Conclusion

Understanding the pathophysiological mechanisms of post–COVID-19 manifestations that affect the ANS is mandatory to look for targeted therapeutic management. It is necessary that all different specialists know how to recognize post-acute COVID-19 dysautonomia to start pharmacological and non-pharmacological interventions that improve the quality of life of these patients and reduce the socioeconomic impact. The potential POTS association with COVID-19 vaccination seems weaker than with COVID-19 infection, but the prevalence of POTS post-vaccination will be clearer as more data are available. The length of POTS related to COVID-19 and the vaccine remains unknown. It is necessary to evaluate the impact of successive reinfections on these patients.

## Author contributions

EG-M, JR-C, and FP-M: conceived, structured, organized this review, and wrote the original draft. DG, JR, AV, FE, GG, MM, KT-H, AM-R, MR-C, and MJ-N: reviewed the literature, organized information compiled, and reviewed and edited the original draft. All authors have read and agreed to the current version of the manuscript, read, approved the final manuscript, and agreed to be accountable for the content of the manuscript.
